# Plasma Midregional Pro-Adrenomedullin Improves Prediction of Functional Outcome in Ischemic Stroke

**DOI:** 10.1371/journal.pone.0068768

**Published:** 2013-07-22

**Authors:** Thomas Seifert-Held, Thomas Pekar, Thomas Gattringer, Nicole E. Simmet, Hubert Scharnagl, Christoph Bocksrucker, Christian Lampl, Maria K. Storch, Tatjana Stojakovic, Franz Fazekas

**Affiliations:** 1 Department of Neurology, Medical University of Graz, Graz, Austria; 2 University of Applied Sciences Wiener Neustadt, Wiener Neustadt, Austria; 3 Clinical Institute of Medical and Chemical Laboratory Diagnostics, Medical University of Graz, Graz, Austria; 4 Konventhospital Barmherzige Brueder Linz, Linz, Austria; 5 Konventhospital Barmherzige Schwestern Linz, Linz, Austria; University of Queensland, Australia

## Abstract

**Background:**

To evaluate if plasma levels of midregional pro-adrenomedullin (MR-proADM) improve prediction of functional outcome in ischemic stroke.

**Methods:**

In 168 consecutive ischemic stroke patients, plasma levels of MR-proADM were measured within 24 hours from symptom onset. Functional outcome was assessed by the modified Rankin Scale (mRS) at 90 days following stroke. Logistic regression, receiver operating characteristics (ROC) curve analysis, net reclassification improvement (NRI), and Kaplan-Meier survival analysis were applied.

**Results:**

Plasma MR-proADM levels were found significantly higher in patients with unfavourable (mRS 3–6) compared to favourable (mRS 0–2) outcomes. MR-proADM levels were entered into a predictive model including the patients' age, National Institutes of Health Stroke Scale (NIHSS), and the use of recanalization therapy. The area under the ROC curve did not increase significantly. However, category-free NRI of 0.577 (p<0.001) indicated a significant improvement in reclassification of patients. Furthermore, MR-proADM levels significantly improved reclassification of patients in the prediction of outcome by the Stroke Prognostication using Age and NIHSS-100 (SPAN-100; NRI = 0.175; p = 0.04). Kaplan-Meier survival analysis showed a rising risk of death with increasing MR-proADM quintiles.

**Conclusions:**

Plasma MR-proADM levels improve prediction of functional outcome in ischemic stroke when added to the patients' age, NIHSS on admission, and the use of recanalization therapy. Levels of MR-proADM in peripheral blood improve reclassification of patients when the SPAN-100 is used to predict the patients' functional outcome.

## Introduction

Ischemic stroke is among the leading causes of death and disability and utilises a huge amount of health care expenses. Clinical criteria which predict worse functional outcome include increased age and higher National Institutes of Health Stroke Scale (NIHSS) on admission. [1] Early pharmacological recanalization improves outcome compared to placebo treatment. [2] A potential biomarker should provide predictive information in addition to established prognostic criteria. [3] Several proteins in peripheral blood which are related to an acute stress response have recently been shown to improve outcome prediction in ischemic stroke. [4]–[7] As derived from observations in patients with myocardial infarction and congestive heart failure (CHF), plasma midregional pro-adrenomedullin (MR-proADM) is an independent predictor of death. [8], [9] We hypothesized that MR-proADM would also reflect the acute stress response in ischemic stroke and could therefore be used to predict functional outcome. MR-proADM is a non-functional precursor of adrenomedullin. [10] This protein has been originally isolated from pheochromocytoma and is found in different organs and tissues including vascular smooth muscle cells and endothelium. [11]–[13] Thereby, it exerts vasodilating, vasoprotective and angiogenic effects. [14] Adrenomedullin is difficult to measure in peripheral blood because of complex formation and rapid clearance from the circulation. [15], [16] The more stable MR-proADM is secreted in equimolar amounts to adrenomedullin and can be reliably detected in human plasma. [17], [18].

## Methods

### Ethics statement

The study was approved by institutional review boards of the Medical University of Graz and Konventhospital Barmherzige Brueder Linz. Written informed consent was obtained from all participants. For patients with impaired consciousness or aphasia, written informed consent was obtained when these patients regained the ability to communicate.

### Patients

Consecutive patients admitted between September 2010 and June 2012 to stroke units of the Departments of Neurology, Medical University of Graz and Konventhospital Barmherzige Brueder Linz, were considered for participation in this study. Patients with acute hemispheric, cerebellar or brainstem ischemia according to clinical examination and brain imaging (computerized tomography or magnetic resonance imaging) were eligible when they had a NIHSS [19] of more than 3 on admission and a modified Rankin Scale (mRS) [20] of 0 or 1 before symptom onset. Blood sampling for this study had to be performed within 24 h from symptom onset and before initiation of recanalization therapy (intravenous or intraarterial thrombolysis, endovascular thrombectomy). Subjects with minor stroke (NIHSS <3), transitory ischemic attack (TIA) or evidence for infectious disease on admission were not included. Patients were not eligible when they had major surgery or transfusion of blood components within one month prior to their stroke. Further exclusion criteria were applied as follows: acute renal failure, acute myocardial infarction, chronic hemodialysis, CHF New York Heart Association (NYHA) classes III and IV, active malignancy, immunosuppressive therapy.

### Clinical variables and laboratory procedure

The NIHSS was obtained on admission by board certified neurologists. The mRS at day 90 following stroke was obtained during a routine follow-up visit or by telephone interviews with patients or their caregivers. [21] The Stroke Prognostication using Age and NIHSS (SPAN) was obtained by combining the patients' age in years and NIHSS on admission. [22] Individuals with SPAN >100 were considered SPAN-100 positive, and those with SPAN <100 were SPAN-100 negative. In a recent analysis, SPAN-100 positivity was associated with a significant lower odds of a composite favourable outcome (mRS <1, NIHSS <1, Barthel index >95, Glasgow Outcome Scale score 1) at three months following stroke after adjusting for thrombolytic treatment. [22] Stroke was classified according to the Oxfordshire Community Stroke Project (OCSP) [23] and the Causative Classification of Stroke System (CCS). [24] Cerebrovascular risk factors were identified as defined by preadmission history or the need for medication at discharge: hypertension, hypercholesterolaemia, and diabetes mellitus. Atrial fibrillation was diagnosed either by history, an electrocardiogram (ECG) on admission, or Holter-ECG during the hospital stay. Clinical care was performed according to guidelines of the European Stroke Organisation. Blood was drawn by venipuncture and collected into EDTA-coated tubes. Plasma was stored at −70°C for further analysis. Plasma MR-proADM was measured by a commercial chemoluminescence assay on a KRYPTOR® system (Thermo Scientific B^.^R^.^A^.^H^.^M^.^S, Hennigsdorf, Germany). [18] Measurements were performed blinded to all clinical data.

### Statistical analysis

Student's t-test, Mann-Whitney's U-test, the Chi-square test or Fisher's exact test, and Spearman's rank order correlation were applied for two-group comparisons. Backwards elimination logistic regression was performed to generate predictive models for functional outcome at day 90 following stroke. Patients were dichotomized into favourable (mRS 0–2) and unfavourable (mRS 3–6) outcomes. From a previous small exploratory study, a sample size of 146 patients was derived to obtain significantly different MR-proADM levels between these patient groups with α = 0.05 and 80% power. To evaluate the added predictive ability of MR-proADM, discrimination of models was assessed by comparing areas under receiver operating characteristics (ROC) curves (AUC) [25] and category-free net reclassification improvement (NRI) [26] was applied. NRI offers incremental information over the comparison of AUCs of ROC curves. [27] Category-free NRI is not influenced by correct scaling of the model and offers the widest and most standardized application in quantification of improvement. [26] Based on outcome prediction by the use of the SPAN-100, categorial NRI was obtained by reclassification of patients according to their plasma MR-proADM quintiles. SPAN-100 negative patients in the upper three quintiles were reclassified upwards. SPAN-100 positive patients in the lower two quintiles were reclassified downwards. Z-statistics were calculated as described previously, [27] and p-value was obtained by GraphPad software. Comparison of ROC curves and Kaplan-Meier analysis were done with MedCalc 11.6.1. and sample size calculation with G*Power 3.1. [28] Other analyses were performed by IBM SPSS Statistics version 20 and R version 2.15.1.

## Results

168 patients were included in the study, 85 men and 83 women at a mean age of 72.9 years (median 74; range 18–97), all of them Caucasians. Blood samples were collected within 12 hours in 138 (82.1%) and between 12 and 24 hours in 30 (17.9%) patients. 90 (53.6%) patients received revascularization therapy. Patients had a median NIHSS of 9 (range 4–25) on admission. No significant differences in the NIHSS were found between patients who received recanalization therapy and them who didn't. No significant differences in the NIHSS were found between men and women, and whether patients had blood sampling within 12 hours from symptom onset or afterwards. Stroke was classified according to the OCSP as follows: 19.6% total anterior circulation syndrome (TACS), 60.2% partial anterior circulation syndrome (PACS), 13.1% posterior circulation syndrome (POCS) and 7.1% lacunar syndrome (LACS). Causes of stroke according to the CCS were found as follows: 33.9% supra-aortic atherosclerosis, 43.5% cardio-aortic embolism, 7.1% small artery occlusion, 15.5% uncommon/undetermined causes. Cerebrovascular risk factors were found as follows: hypertension in 130 (77.4%), hypercholesterolemia in 86 (51.2%), diabetes mellitus in 40 (23.8%), and atrial fibrillation in 68 (40.5%) patients. Correlations of plasma MR-proADM were found with the patients' age (r_S_ = 0.34; p<0.001), NIHSS on admission (r_S_ = 0.18; p = 0.023) and mRS at day 90 (r_S_ = 0.33; p<0.001). MR-proADM levels did not significantly differ between men and women, between patients with diabetes mellitus or without, and between patients with hypercholesterolemia or without. Higher median MR-proADM levels were found in patients with hypertension (0.77 vs. 0.64 nmol/l; p = 0.005), atrial fibrillation (0.85 vs. 0.70 nmol/l; p<0.001) and coronary heart disease (0.82 vs. 0.74 nmol/l; p = 0.041).

**Figure 1:**
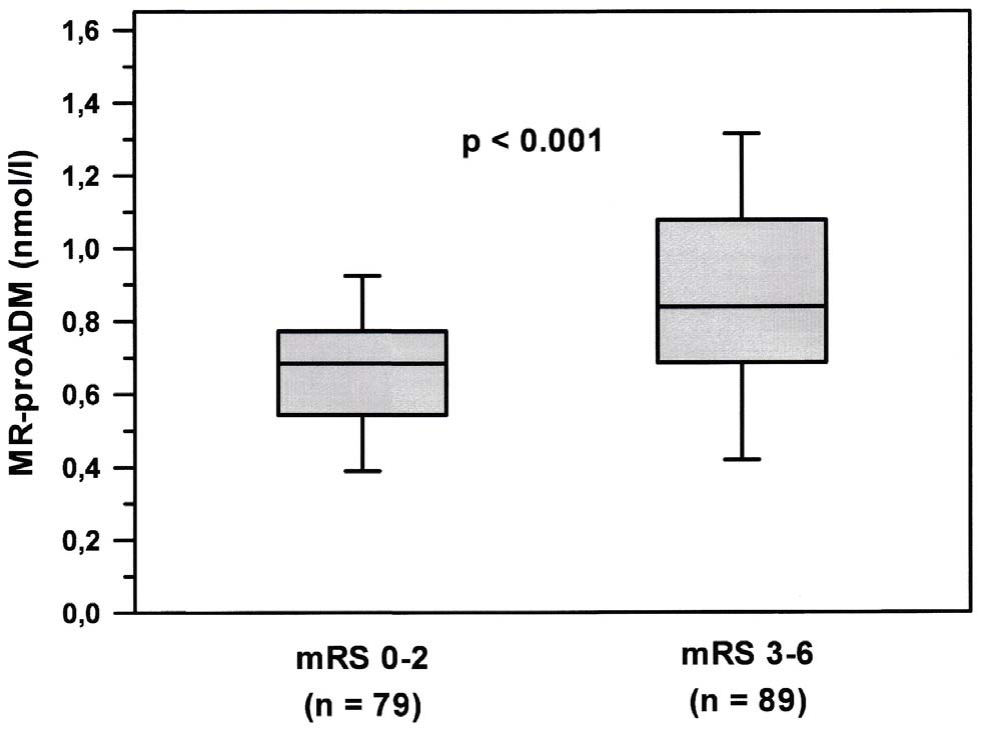
Plasma midregional pro-adrenomedullin (MR-proADM) levels in patients on admission. Patients were dichotomized into favourable (mRS 0–2) and unfavourable (mRS 3–6) outcomes at day 90 after stroke. Plots display the median, interquartile range (box), 10^th^ and 90^th^ percentiles (whiskers). Abbreviation: mRS  =  modified Rankin Scale; MR-proADM  =  midregional pro-adrenomedullin.

Patients had a median mRS of 3 at day 90. Patients with unfavourable outcome were significantly older, had a higher median NIHSS, a higher proportion of TACS and a higher prevalence of atrial fibrillation and cardio-aortic embolism as the cause of stroke (table 1). Plasma MR-proADM was found significantly higher in patients with unfavourable compared to favourable outcomes (median 0.84 vs. 0.68 nmol/l; p<0.001; figure 1). Predictive models were generated to assess the value of adding MR-proADM plasma levels to the patients' age, NIHSS on admission, and the use of recanalization therapy (table 2). The AUC of generated ROC curves did not increase significantly when plasma MR-proADM levels were added (0.803 and 0.819 for models 1 and 2, respectively; p = 0.204). Category-free NRI of 0.577 (p<0.001) indicated a significant improvement in reclassification of patients by adding MR-proADM levels to predict functional outcome.

**Table 1:** Demographic data and baseline clinical characteristics of patients.

	all patients	RS 0–2	mRS 3–6	p
n	168	79	89	–
**median age (range)**	74 years (18–97)	71 years (18–97)	79 years (60–89)	<0.001
**male : female**	85 : 83	43 : 36	42 : 47	0.434
**median NIHSS on admission**	9 (range 4–25)	6	12	<0.001
**recanalization therapy**	90 (53.6%)	46 (58.2%)	44 (49.4%)	0.325
**hypertension**	130 (77.4%)	59 (74.7%)	71 (79.8%)	0.547
**hypercholesterolemia**	86 (51.2%)	50 (63.3%)	36 (40.4%)	0.005
**diabetes mellitus**	40 (23.8%)	19 (24.1%)	21 (23.6%)	0.911
**atrial fibrillation**	68 (40.5%)	18 (22.8%)	50 (56.2%)	<0.001
**coronary heart disease**	30 (17.9%)	11 (13.9%)	19 (21.3%)	0.293
**angiotensin convertingenzyme inhibitors**	42 (25.0%)	16 (20.3%)	26 (29.2%)	0.246
**angiotensinreceptor antagonists**	21 (12.5%)	8 (10.1%)	13 (14.6%)	0.520
**TACS**	33 (19.6%)	5 (6.3%)	28 (31.5%)	<0.001
**PACS**	101 (60.2%)	55 (69.6%)	46 (51.7%)	0.044
**POCS**	22 (13.1%)	10 (12.7%)	12 (13.5%)	0.943
**LACS**	12 (7.1%)	8 (10.1%)	4 (4.5%)	0.265
**supra–aortic atherosclerosis**	57 (33.9%)	24 (30.4%)	33 (37.1%)	0.452
**cardio-aortic embolism**	73 (43.5%)	26 (32.9%)	47 (52.8%)	0.025
**small artery occlusion**	12 (7.1%)	9 (11.4%)	3 (3.4%)	0.086
**uncommon/undetermined causes of stroke**	26 (15.5%)	17 (21.5%)	9 (10.1%)	0.068

Patients were dichotomized into favourable (mRS 0–2) and unfavourable (mRS 3–6) outcomes at day 90 after stroke. P-values for median age and median NIHSS on admission were obtained by Mann-Whitney's U-test. Other p-values were obtained by the Chi-square test or Fisher's exact test.

**Table 2:** Predictive models for an unfavourable functional outcome (modified Rankin Scale 3–6) at day 90 following stroke.

	variables	OR (95% CI)	p
**model 1** a	age	1.097 (1.057–1.139)	<0.001
	NIHSS	1.193 (1.108–1.284)	<0.001
	recanalization therapy	0.587 (0.277–1.245)	0.160
**model 2** a	age	1.090 (1.049–1.132)	<0.001
	NIHSS	1.187 (1.100–1.280)	<0.001
	recanalization therapy	0.732 (0.332–1.615)	0.439
	plasma MR-proADM	4.062 (1.109–14.87)	0.028

Abbreviations: NIHSS  =  National Institutes of Health Stroke Scale; OR  =  Odd's ratio; CI  =  confidence interval.

a Areas under receiver operating characteristics (ROC) curves (AUC) 0.803 and 0.819 for models 1 and 2, respectively (p = 0.204); category-free net reclassification improvement (NRI) 0.577 (p<0.001).

Abbreviations: mRS  =  modified Rankin Scale; NIHSS  =  National Institutes of Health Stroke Scale; TACS  =  total anterior circulation syndrome; PACS  =  partial anterior circulation syndrome; POCS  =  posterior circulation syndrome; LACS – lacunar syndrome.

With plasma MR-proADM levels in the upper three quintiles, 100%, 83.3% and 75.5% of patients had an unfavourable functional outcome, respectively, as compared to 36.4% and 47.4% of patients in the lower two quintiles. When using the SPAN-100 for outcome prediction (table 3), in 36 patients with unfavourable outcome reclassification improved by MR-proADM, and in 8 patients it became worse, with a net gain in reclassification proportion of 0.31 (p<0.001). Eleven individuals with favourable outcome were falsely reclassified by MR-proADM quintiles (p<0.01). Overall NRI was 0.175 (p = 0.04) indicating an improvement in reclassification of patients by adding plasma MR-proADM to their SPAN-100 status.

**Table 3:** Reclassification table for prediction of functional outcome at day 90 following stroke.

		SPAN-100+ MR-proADM, predicted outcome		
		mRS 0–2	mRS 3–6	total
**SPAN-100, predicted outcome**	**events (observed outcome mRS 3–6)**			
	mRS 0–2	33	36	69
	mRS 3–6	8	12	20
	total	41	48	89
	**non-events (observed outcome mRS 0–2)**			
	mRS 0–2	66	11	77
	mRS 3–6	0	2	2
	total	66	13	79

Reclassification was performed using the SPAN-100 alone or in combination with MR-proADM quintiles.

Abbreviations: SPAN  =  Stroke Prognostication using Age and NIHSS; MR-proADM  =  midregional pro-adrenomedullin; mRS  =  modified Rankin Scale.

**Figure 2:**
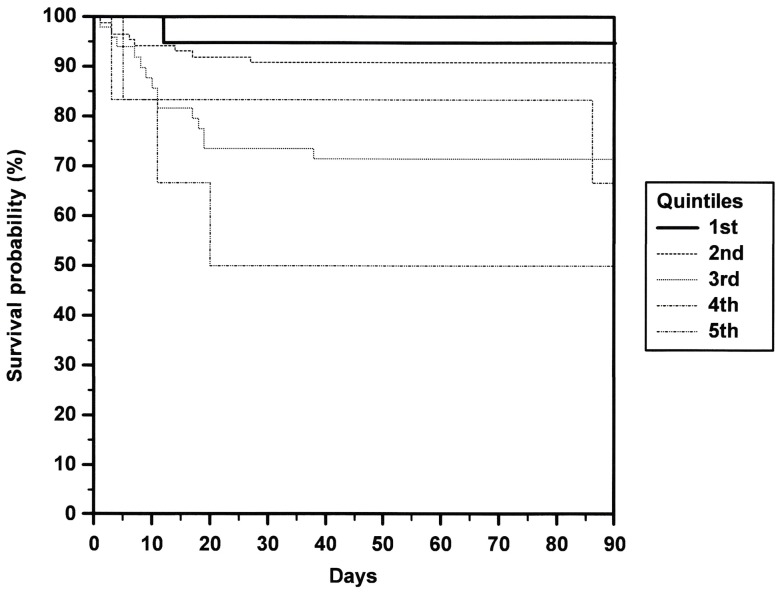
Kaplan-Meier survival curves. Time to death related to plasma MR-proADM quintiles (1^st^: 0.04–0.45 nmol/l; 2^nd^: 0.46–0.86 nmol/l; 3^rd^: 0.87–1.27 nmol/l; 4^th^: 1.28–1.68 nmol/l; 5^th^: 1.69–2.10 nmol/l).

Patients who died within 90 days following stroke (n = 30) had a higher median NIHSS (15 vs. 7; p<0.001) and higher MR-proADM levels (median 0.92 nmol/l vs. 0.73 nmol/l; p<0.001). The difference in age compared to patients who survived did not reach statistical significance (median 79.0 vs. 73.0 years; p = 0.058). In models to predict the patients' death within 90 days following stroke, the AUC of generated ROC curves did not increase significantly when plasma MR-proADM levels were added to the patients' age, NIHSS and the use of recanalisation therapy (data not shown). Category-free NRI of 0.127 (p = 0.523) showed no improvement in reclassification of patients by adding MR-proADM levels to predict patients' death. Kaplan-Meier survival analysis showed a rising risk of death with increasing MR-proADM quintiles (p = 0.011; figure 2).

## Discussion

Plasma MR-proADM improves prediction of functional outcome in ischemic stroke when added to the patients' age, NIHSS on admission, and the use of recanalization therapy. Levels of MR-proADM in peripheral blood improve reclassification of patients when the SPAN-100 is used in the prediction of functional outcome. Currently, there are no commonly accepted models to predict functional outcome in ischemic stroke. The SPAN-100 has a great advantage in its ease of use in clinical routine and emergency settings. [29] The mRS at three months is the most prevalent outcome assessment and the preferred outcome measure for treatment trials in acute stroke. [30], [31] As opposed to other stroke biomarker studies, [4]–[6], [32], [33] we have excluded patients with minor stroke or transitory ischemic attack which results in a higher median NIHSS in our study. This contributes to the higher percentage of patients in this study who underwent recanalization therapy as compared to average rates in Austrian stroke units in recent years. [34] We could not approach all eligible patients in the given timeframe for participation in the study. However, we included patients consecutively according to the aforementioned criteria irrespective of any clinical prediction of their prognosis and did not include patients with preexisting disability.

Adrenomedullin has been identified as a tumor survival factor [35] and exerts antimicrobial properties. [36] We have excluded patients with a known malignancy or with signs of infection. In patients with myocardial infarction or CHF, plasma MR-proADM is an independent predictor of death. [8], [9] In our study, Kaplan-Meier survival analysis showed a rising risk of death with increasing plasma level quintiles. MR-proADM levels have previously shown to increase with higher NYHA classes. [9] In that study, MR-proADM appeared to decrease with the intake of angiotensin-converting enzyme (ACE) inhibitors or angiotensin receptor antagonists. [9] In our cohort, we have excluded patients with NYHA classes III and IV. The proportions of patients who were on ACE inhibitor or angiotensin receptor antagonist therapy in our study did not differ in patients with favourable (mRS 0–2) and unfavourable (mRS 3–6) outcomes (table 1). Patients who took either an ACE inhibitor or an angiotensin receptor antagonist on admission had a higher median mRS at day 90 (4 vs. 2; p = 0.015) and higher median MR-proADM levels (0.82 vs. 0.70 nmol/l; p<0.001).

Adrenomedullin is supposed to counter vasoconstricting and sodium-retaining hormones in patients with CHF. [37] The counter-regulation of vasoconstriction as part of a systemic stress response may also apply to patients with acute ischemic stroke. Data from animal models hint to a role of adrenomedullin in neuroprotection, [38]–[40] an issue to be addressed in future clinical trials. The findings from our exploratory study show that the determination of MR-proADM levels in peripheral blood improves prediction of functional outcome in ischemic stroke patients. This should be reassessed in a larger trial to evaluate its applicability in routine clinical procedures.
